# Intestinal Epithelial Organoids as Tools to Study Epigenetics in Gut Health and Disease

**DOI:** 10.1155/2019/7242415

**Published:** 2019-01-27

**Authors:** Judith Kraiczy, Matthias Zilbauer

**Affiliations:** ^1^Department of Paediatrics, University of Cambridge, Cambridge, UK; ^2^Wellcome Trust-Medical Research Council Stem Cell Institute, University of Cambridge, Cambridge, UK

## Abstract

The intestinal epithelium forms the inner layer of the human intestine and serves a wide range of diverse functions. Its constant exposure to a vast amount of complex microbiota highlights the critical interface that this single-cell layer forms between the host and our environment. Importantly, the well-documented contribution of environmental factors towards the functional development of the human intestinal epithelium directly implies epigenetic mechanisms in orchestrating this complex interplay. The development of intestinal epithelial organoid culture systems that can be generated from human tissue provides researchers with unpresented opportunities to study functional aspects of human intestinal epithelial pathophysiology. In this brief review, we summarise existing evidence for the role of epigenetics in regulating intestinal epithelial cell function and highlight the great potential for human gut organoids as translational research tools to investigate these mechanisms *in vitro*.

## 1. Introduction

The intestinal epithelium serves a large variety of diverse functions including absorption of nutrients and water as well as forming a critical barrier to the environment [1]. The latter requires the constant crosstalk between host cells with luminal contents as well as a variety of immune cells located in the underlying mucosa. Robust evidence has highlighted the requirement of environmental factors (both host internal and external) towards driving functional development of the intestinal epithelium. The ability to mediate exposure to environmental factors into potentially stable alterations of cellular function is a hallmark of all epigenetic mechanisms [2]. Hence, their involvement in regulating cellular function of the intestinal epithelium during development and in healthy homeostasis follows as a logical conclusion. Moreover, epigenetic mechanisms are increasingly being recognised as the missing link between environmental triggers and the rising incidence of several chronic noncommunicable diseases including those that affect or originate from the gastrointestinal (GI) tract [3]. Despite the plausible concept of epigenetics as mediator of the crosstalk between environment and cellular function, providing direct evidence has proven to be challenging. Given the complexity of environmental factors that might contribute to shaping the intestinal epithelial epigenome *in vivo*, a reductionist approach may be beneficial in order to identify underlying mechanisms. In this respect, the development of three-dimensional organoid models, which closely resemble the *in vivo* situation, has provided unprecedented opportunities for scientists to investigate fundamental aspects of cell biology. Importantly, the ability to generate such organoids from human tissues further highlights the value of these models as translational research tools. In this short review, we will briefly summarise current evidence supporting a key role for epigenetic mechanisms with a focus on DNA methylation in regulating cellular function of the intestinal epithelium and highlight the value of human gut organoid models as translational research tools to investigate these mechanisms in vitro.

### 1.1. Epigenetic Mechanisms

Epigenetics can be defined as any mechanism leading to a potentially heritable change in cellular phenotype without altering the underlying DNA sequence [2–4]. Main epigenetic mechanisms currently known to be operative in mammals include (i) chromatin structure, (ii) posttranslational modifications of histones, (iii) expression of noncoding RNAs, and (iv) DNA methylation. Briefly, posttranslational modifications (PTMs) of histone tails can alter chromatin structure and DNA accessibility, thereby impacting on gene transcription and ultimately cellular function [5]. PTMs include phosphorylation, methylation, and acetylation, which can lead to either silencing or activation of associated genes [5]. In contrast, expression of noncoding RNAs (ncRNAs) such as microRNAs (miRs) and long noncoding RNAs (lncRNAs) regulates gene transcription by degrading their target mRNAs or preventing their translation [6]. A single miRNA can have multiple mRNA targets thereby being capable of influencing complex cellular pathways [6]. Lastly, DNA methylation refers to the addition of a methyl group to the cytosine in the DNA, which in mammals occurs mostly in the context of a cytosine-guanine (C-G) sequence (CpG) [7]. Although our understanding of how DNA methylation regulates gene transcription remains incomplete, the impact on transcription factor accessibility and binding affinity to gene promoters—either directly or via the recruitment of methyl-CpG binding domain (MBD) proteins—has been well documented. Furthermore, methylation may physically affect the DNA by altering its mechanical properties [8]. DNA methylation is catalyzed by a class of enzymes named DNA methyltransferases (DNMTs), which require a methyl donor molecule. While Dnmt1 is traditionally considered the “maintenance methyltransferase” [9], Dnmt3a and Dnmt3b, as well as Dnmtl, have been implicated in mainly establishing “de novo” methylation marks. [10, 11]. The removal of DNA methylation marks is a complex process and has been found to be partly regulated by ten-eleven translocation (TET) family enzymes, member 1-3 (Tet1-3) [12–15]. Tet1 prevents hypermethylation throughout the genome, hereby acting as a maintenance demethylase [16, 17].

The interplay between the various epigenetic mechanisms highlights the complexity of cellular regulatory networks and the need to develop suitable experimental approaches to unravel their implication in health and disease. Furthermore, the stability—or potential reversibility—of epigenetic marks is a critical factor both with regards to the impact on disease development as well as in light of novel treatment approaches aimed at reversing disease associated molecular changes.

A developmental origin of disease has been proposed for many multifactorial, complex diseases [18]. At the heart of this concept is the long-term exposure to certain environmental factors particularly during critical, more susceptible time periods. Considering the importance of epigenetic mechanisms in regulating cellular development, combined with their responsiveness to environmental factors, implicates these mechanisms directly into the conceptual framework of disease development.

### 1.2. Human Intestinal Organoid Models

Human intestinal epithelial organoids (IEOs) are self-organizing, three-dimensional structures that can be propagated long term and differentiated into all different epithelial cell subsets [19, 20]. IEOs can be generated either from pluripotent stem cells, such as embryonic or induced pluripotent stem cells (iPSC), or by expanding adult intestinal stem cells [21]. A key expression marker of the latter is leucine-rich repeat-containing G protein-coupled receptor 5 (Lgr5) [22]; identification of which can be considered as a major breakthrough in the development of gut organoids [19, 20, 23]. Whilst Lgr5+ adult stem cell-derived organoids give rise to cultures that exclusively consist of intestinal epithelial cells, pluripotent stem cell-derived organoids may also contain mesenchymal cells [24, 25]. However, modifications in culturing protocols also allow to generate epithelial cell-specific intestinal organoids from iPSC [26–28].

In recent years, an increasing number of groups have successfully established human IEO culture systems, and as a result, the field has seen major progress. Several studies have provided compelling evidence that organoids closely mimic *in vivo* structure and cellular dynamics. Whilst the majority of these studies focused on small intestinal IEO, the use of other gut regions like the colon is emerging. Importantly, it could be shown that IEOs retain distinct gut segment-specific phenotypic differences and expression profiles that closely resemble the tissue they were derived from [29–31]. Similarly, a number of elegant studies have shown that iPSC can be successfully differentiated to closely mimic phenotype and gene expression of human colonic epithelium [28, 32, 33]. More recently, work from our group and others have started to investigate the use of human IEOs as translational research tools to explore the role of epigenetic mechanisms in GI development, healthy homeostasis, and related diseases [30, 34].

## 2. Epigenetics in Human Intestinal Epithelial Cell Development

The mature adult intestinal epithelium is a highly dynamic, polarized, single-cell layer that forms the most inner lining of the intestinal mucosa. Its diverse functions include nutrient absorption, water retention, barrier function, antigen sampling, and maintaining immune homeostasis. In order to meet these requirements, the intestinal epithelial cell (IEC) layer is composed of six differentiated epithelial cell subtypes: enterocytes, goblet cells, enteroendocrine cells, tuft cells, M-cells, and small intestinal Paneth cells [35]. All epithelial cell subtypes are derived from intestinal stem cells (ISCs), which are located in a “niche” environment at the bottom of crypts in both the small and large intestine [35–37]. These mostly rapidly dividing, Lgr5 expressing cells are therefore responsible for the constant replenishment of the epithelial cell layer, which regenerates over approximately 3-4 days [22].

As the most inner lining of the GI tract, the intestinal epithelium is constantly exposed to a multitude of external factors including food antigens and the diverse microbiota [1]. From early fetal to adult life, these environmental factors themselves undergo substantial changes and are thought to be essential for the structural as well as functional development of the intestinal epithelium [38–42]. During the first months after birth, the infant microbial composition is highly dynamic and under the influence of nutritional factors such as breast- vs formula feeding and reaches a settled state after 1-2 years [42]. On the background of an assumed stable genome throughout the lifespan, environmentally driven changes to phenotype imply that epigenetic mechanisms are operative. Indeed, a number of elegant studies have provided compelling support for this concept.

Evidence for the importance of the early postnatal time window and specifically requirement for bacterial colonization was provided by studies using germ-free mice. The authors were able to demonstrate substantial differences in intestinal epithelial DNA methylation between germ-free and conventionally raised mice, which were found to be most prominent during the immediate postnatal period [43, 44]. Pattern recognition receptors, such as Toll-like receptors, are essential components of the intestinal innate immune defence, as they are able to sense bacterial products and mount an adequate response. As an example, studies by Takahashi et al. provided evidence for the epigenetically regulated expression and function of TLR4 in dependence of bacterial colonization of the large intestine [45, 46]. In contrast, it was shown that microbial colonization did not affect the chromatin landscape but induced strong transcriptional changes [47]. Investigating these concepts in humans is more challenging and highlights the great value of intestinal organoids as will be outlined below. However, work from our own group using primary human epithelial cell samples has provided support for the importance of DNA methylation in regulating human intestinal epithelial cell function in the transition from fetal to paediatric epithelium [48]. We performed simultaneous genome-wide DNA methylation and gene expression analyses on purified primary intestinal epithelium obtained from human fetal gut and paediatric biopsies. IEC DNA methylation was found to be highly age- and gut segment specific with substantial developmental methylation changes being associated with differences in gene expression. Importantly, gene ontology analyses of genes with dynamic DNA methylation and gene expression changes revealed a significant enrichment for cellular development as well as immunological and gastrointestinal disease. The latter suggests that alterations in epigenetic programming may predispose to the development of inflammatory bowel diseases (IBD) [18].

The ability to derive intestinal organoids from human fetal as well as adult gut samples combined with iPSC/ESC-derived organoid models has opened up novel opportunities to study human IEC development in vitro [27, 49–51]. In recent work, our group has generated genome-wide DNA methylation profiles from IEOs derived from human fetal gut and paediatric biopsies. Comparing with epigenetic profiles derived from matching primary purified epithelial cell samples, we were able to demonstrate that organoids retain their regional epigenetic signatures in culture [30]. Moreover, we observed striking DNA methylation changes in fetal organoids over prolonged culture periods. Detailed analyses revealed that these changes seemed to represent a degree of in vitro maturation, a process, which was partly abrogated by ablation of the demethylating enzyme TET1 using CRISPR/Cas9 technology. In contrast, DNA methylation signatures of organoids derived from paediatric or adult mucosal biopsy samples were found to be much more stable over prolonged culture periods. Together, these findings not only provide further support for human fetal gut organoids as highly promising tools but also confirm significant changes in epigenetic plasticity between human fetal and adult epithelium.

## 3. Epigenetics in Intestinal Epithelial Homeostasis

As mentioned above, in the healthy adult intestinal epithelium, rapidly cycling stem cells give rise to all epithelial cell subsets as daughter cells which migrate up the crypt villus axis [22]. Gene expression and cellular function of epithelial cell subsets vary substantially, ranging from the production of antimicrobial peptides in Paneth cells, over mucin proteins in goblet cells to hormones secreted by enteroendocrine cells. Hence, distinct phenotypic changes occur during the differentiation process on a stable genetic background, implying the possibility of epigenetic mechanisms in contributing towards these processes [52].

Indeed, cellular differentiation from stem cell to specialized cell types has been shown to involve processes of epigenetic remodelling. Several studies performed in mice that compared DNA methylomes of crypt versus villus epithelial cells discovered distinct yet overall relatively minor differences [53–56]. For example, Kaaij and colleagues found very limited number of differentially methylated regions (DMRs) between Lgr5+ stem cell and differentiated villus cells [53]. A similar study also detected DMRs with relatively small changes of magnitude; however, these methylation changes were found to be located at enhancers of proliferation genes that regulate IEC cell division and differentiation [54].

Other groups have taken a different approach by investigating the effect of IEC-specific Dnmt1 ablation in mice from birth, which was found to be associated with aberrant epithelial differentiation, increased apoptosis, and DNA damage resulting in postnatal lethality [57]. Interestingly, IEC-specific deletion of DNMT1 in the adult intestinal epithelium led to aberrant crypt fission and expansion with increased Lgr5 expression [54]. The retained viability of these mice despite lack of such a critical enzyme was thought to be compensated by upregulation of Dnmt3b. Indeed, simultaneous deletion of both DNA methyltransferases DNMT1 and DNMT3B destroyed crypt-villus organization and lead to reduced survival [58]. Furthermore, Dnmt1 was shown to be required for intestinal organoid establishment, but not required for their maintenance [57]. Alongside DNA methylation marks, the role of DNA hydroxymethylation (hmC) is increasingly being recognized. An in vitro study has shown that hmC is increasing in differentiating epithelial cells, specifically at transcription factor binding sites of differentiation genes [59]. In a mouse model, hmC marks were shown to be preferably gained at genes that also increase in expression during differentiation such as intestinal alkaline phosphatase (Alpi). Mice lacking Tet1 in the intestinal epithelium consequently showed a reduced number of Lgr5+ stem cells and reduced organoid-forming capacity [60]. This observation is comparable to the reduced culturing capacity of human intestinal organoids with disrupted TET1 [30].

In addition to DNA methylation, the role of PTH marks in gene regulation of the intestinal epithelium has gained increasing interest. A landmark study investigated activating histone mark patterns (H3K27ac and H3K4me2) during the process of differentiation of intestinal stem cells towards enterocyte—or secretory precursors. Notably, the tested marks remained overall similar between those cell types, allowing for remarkable plasticity between those lineage precursors [61]. Similarly, distribution of the silencing H3K27me3 mark was overall unchanged between crypt and villus compartments [62]. Chromatin accessibility, however, was shown to change selectively to control expression of lineage-restricted genes [63].

In light of the above, one may speculate that in the absence of major epigenetic remodelling, the underlying epigenetic programme of intestinal epithelial stem cells may mediate their response to signals from the microenvironment. Indeed, using human intestinal organoids derived from different gut segments, our group was able to show that during an in vitro differentiation (by withdrawal of Wnt agonists), the underlying epigenetic DNA methylation profile determined inducibility of gut segment specific genes. Indeed, hypomethylation induced by coculture of small bowel organoids with DNMT inhibitors led to the induction of colonic epithelial cell markers [30].

Together these findings illustrate how epigenetic marks are critical for the maintenance of tissue and cell type-specific cell function. Importantly, organoid models have shown to provide an elegant tool to address these fundamental questions of human intestinal cell biology.

## 4. Epigenetics in IEC Malfunction and Disease

Epigenetics mechanisms work at the interface between the human genome and our environment [2]. In the context of a changing lifestyle and environment, they thus present a plausible framework for the rising incidence of noncommunicable and complex diseases. With regards to the gastrointestinal (GI) tract, several cancer types have been shown to harbour aberrant DNA methylation signatures [64–71]. For example, in colon cancer, both a global genomic hypomethylation as well as locus-specific hypermethylation have been observed [66, 68, 72]. More specifically, a number of elegant studies have been able to demonstrate how promoter hypermethylation of tumour suppressor genes can initiate tumour growth, possibly in response to long-term exposure to specific environmental factors [72–75].

In addition to cancer, the rapid rise in the incidence of several noncommunicable chronic inflammatory conditions is increasingly being linked to environmental influences and thereby placing epigenetic mechanisms in the spotlight of disease pathogenesis. Amongst these conditions are chronic inflammatory bowel diseases (IBD), which comprise the two main entities, Crohn's disease (CD) and ulcerative colitis (UC). IBD can affect patients at any age but are increasingly being diagnosed in children and young adults [76, 77]. Although our understanding of disease pathogenesis remains incomplete, it is widely accepted that altered function of the intestinal epithelium plays an important role either in causing and/or maintaining chronic mucosal inflammation in IBD patients [78]. As part of recent work, our group performed genome-wide DNA methylation profiling of purified primary intestinal epithelium obtained from children newly diagnosed with IBD and matched healthy controls. Results revealed disease-associated alterations in the epigenetic profile of IBD patients. Importantly, these changes were found to be partly independent of mucosal inflammation and stable over time, with altered DNA methylation levels highly correlated over the course of disease regardless of treatment success or failure [34]. The latter suggest that these epigenetic alterations may indeed contribute towards driving chronic relapsing inflammation in IBD patients. Interestingly, in contrast to these findings, full biopsy specimens from the colon of UC patients showed reversal of epigenetic variation upon mucosal healing [79]. The major impact of differences in cellular composition of mixed cell tissue samples on genome-wide epigenetic profiles are likely explanations for the discrepancies in these studies. Thus, given the complexity of interactions between different cell types present in the intestinal mucosa as well as with their environment (including the gut microbiota), a reductionist approach by using IEO organoid culture model offers major advantages. These include the generation of IEO derived from different gut segments (e.g., affected and nonaffected by the disease) as well as at different stages during the course of disease (e.g., prior to the start and on medication). Importantly, by removing other cell types and the environmental factors, organoid culture systems allow to specifically investigate intestinal epithelial cell intrinsic mechanisms. A number of studies have reported on the use of mucosa-derived IEOs in the context of IBD and provided evidence for patient-derived cultures to retain disease specific alterations in vitro [34, 80–84]. In keeping with these reports, we were able to demonstrate that IBD patient-derived IEOs retain at certain loci their disease-specific DNA methylation signatures in culture [34]. Together, these promising findings strongly support the use of patient-derived IEO as translational research tools to advance our understanding of IBD pathogenesis and to develop improved approaches to manage these conditions.

## 5. Future Perspectives

Major developments in the field of human intestinal organoid culture models have highlighted their value as powerful tools to model intestinal development and healthy homeostasis as well as GI diseases. With regards to furthering our understanding of epigenetic mechanism and how they contribute towards the regulation of these fundamental processes, organoid models offer a number of particular advantages. These include the ability to investigate cell type intrinsic mechanisms in a purely epithelial cell-containing model as well as the option of testing the effect of individual environmental factors on epigenetic signatures by performing specific coculture experiments. The latter option will further benefit from recent advances which have allowed coculture of IEOs with other cell types such as lymphocytes and mesenchyme [85, 86]. Last but not least, the rapid increase in strong evidence supporting the fact that organoids faithfully retain GI disease-specific features in culture emphasises their value for the development of biobanks, drug testing, and drug discovery in the near foreseeable future.
